# Integrated DNA and RNA sequencing reveals early drivers involved in metastasis of gastric cancer

**DOI:** 10.1038/s41419-022-04838-1

**Published:** 2022-04-21

**Authors:** Jieyun Zhang, Fatao Liu, Yanan Yang, Nuoya Yu, Xiaoling Weng, Yue Yang, Zhe Gong, Shenglin Huang, Lu Gan, Sijie Sun, Xiaowei Zhang, Yiwei Gong, Yun Liu, Weijian Guo

**Affiliations:** 1 Department of Gastrointestinal Medical Oncology, Fudan University Shanghai Cancer Center, Shanghai, China; 2 Department of Oncology, Shanghai Medical College, Fudan University, Shanghai, China; 3 Department of General Surgery, Xinhua Hospital, Affiliated to Shanghai Jiao Tong University School of Medicine, Shanghai, China; 4 Shanghai Key Laboratory of Biliary Tract Disease Research, Shanghai, China; 5 Shanghai Research Center of Biliary Tract Disease, Shanghai, China; 6 Ningbo Aitagene Technology Co. LTD, Shanghai, China; 7 Fudan University Shanghai Cancer Center, Key Laboratory of Medical Epigenetics and Metabolism, Institutes of Biomedical Sciences, Fudan University, Shanghai, China; 8 Bio-X Institutes, Key Laboratory for the Genetics of Developmental and Neuropsychiatric Disorders (Ministry of Education), Shanghai Jiao Tong University, Shanghai, China; 9 Institutes of Biomedical Sciences, Fudan University, Shanghai, China; 10 Key Laboratory of Molecular Medicine, The Ministry of Education, Department of Biochemistry and Molecular Biology, Fudan University Shanghai Medical College, Shanghai, China

**Keywords:** Gastric cancer, Cancer genetics

## Abstract

Gastric cancer (GC) is the second cause of cancer-related death and metastasis is an important cause of death. Considering difficulties in searching for metastatic driver mutations, we tried a novel strategy here. We conducted an integrative genomic analysis on GC and identified early drivers lead to metastasis. Whole-exome sequencing (WES), transcriptomes sequencing and targeted-exome sequencing (TES) were performed on tumors and matched normal tissues from 432 Chinese GC patients, especially the comparative analysis between higher metastatic-potential (HMP) group with T1 stage and lymph-node metastasis, and lower metastatic-potential (LMP) group without lymph-nodes or distant metastasis. HMP group presented higher mutation load and heterogeneity, enrichment in immunosuppressive signaling, more immune cell infiltration than LMP group. An integrated mRNA-lncRNA signature based on differentially expressed genes was constructed and its prognostic value was better than traditional TNM stage. We identified 176 candidate prometastatic mutations by WES and selected 8 genes for following TES. Mutated TP53 and MADCAM1 were significantly associated with poor metastasis-free survival. We further demonstrated that mutated MADCAM1 could not only directly promote cancer cells migration, but also could trigger tumor metastasis by establishing immunosuppressive microenvironment, including promoting PD-L1-mediated immune escape and reprogramming tumor-associated macrophages by regulating CCL2 through Akt/mTOR axis. In conclusion, GCs with different metastatic-potential are distinguishable at the genetic level and we revealed a number of potential metastatic driver mutations. Driver mutations in early-onset metastatic GC could promote metastasis by establishing an immunosuppressive microenvironment. This study provided possibility for future target therapy of GC.

## Introduction

Gastric cancer (GC) is the second causes of cancer deaths around the world. In China, it was estimated that 679,100 GC patients were diagnosed each year and most of them were diagnosed at an advanced stage [1]. It is universally accepted that mutations increase in metastasis process, but if tumors gain metastatic ability through accumulation of mutations remains unclear [2]. Some large-scale sequencing did not detect metastatic driver mutations [3–5]. With the development of next-generation sequencing, recent articles discovered some genetic drivers that could partially explain the early development of metastasis [6–10].

The metastatic molecular profile of GC is still unclear. Considering early prometastastic drivers established in primary tumor and passenger mutations accumulating in the metastasis process, traditional comparative analysis between primary and metastasis tumors might lead to misidentification of driver mutations [11–13]. It is necessary to use more appropriate samples and methods to identify metastasis-associated genomic aberrations. We conceived that comparative analysis between primary tumors with different metastatic-potential will help to identify early drivers in primary lesions.

**Fig. 1:**
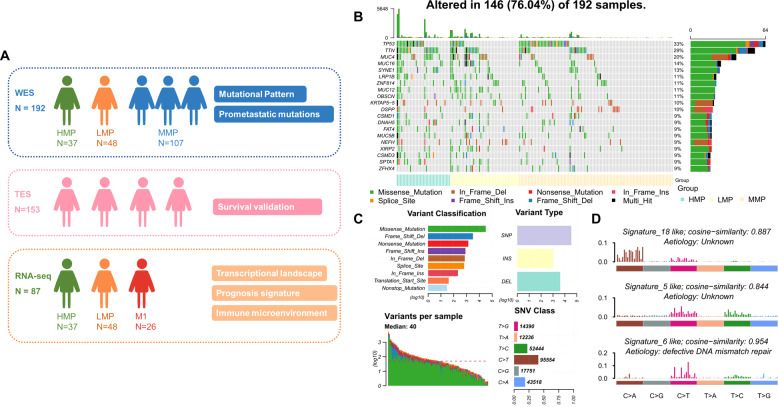
Summary of integrated DNA and RNA sequencing in this study and alterations landscape from WES data of FUSCC GC cohort (*N* = 192). **A** Summary of integrated DNA and RNA sequencing in this study. **B** Landscape of genetic alterations of 192 FUSCC GC patients. The samples were sorted by metastatic-potential and the number of mutations. Top 20 mutated genes were presented which were sorted by mutation rates. **C** Summary of variants across 192 FUSCC GC samples, including variant classification, variant type (SNP/INS/DEL), number of variants and SNV class. **D** Signatures were extracted by decomposing matrix of base substitutions, which were classified into 96 patterns based on mutation type and nucleotides adjacent to the mutated base. The most similar validated signatures were shown and cosine-similarity was calculated to identify best match.

Tumor progression consist of metastasis to regional lymph nodes and dissemination to distant organs. Recently studies confirmed that cancer cells in lymph node involved in dissemination and seeding distant sites [14, 15]. It indicated that patients with lymph node involvement have a tendency to metastasize distantly. Commonly, patients with early GC (EGC) limited to gastric mucosa or submucosa (T1) are less prone to lymph-node metastases [16]. The rate of lymph-node metastasis in EGC was reported from 5.7 to 14% [17–20], and the 10-year survival rate of EGC patients with or without lymph-node metastasis is around 72 or 92%, separately [21]. So we conceived that EGC with lymph-node metastasis (T_1_N_+_M_any_) had higher metastatic-potential (HMP), since metastatic cancer cells occur early in primary tumors instead of accumulating along with the development of metastasis, and be suitable as a model for screening of early prometastastic drivers. Here, we presented the molecular landscape of 432 GC patients characterized by integrated DNA and RNA sequencing, especially the comparative analysis results between HMP group with EGC and lymph-node metastasis (T_1_N_+_M_any_) and lower metastatic-potential (LMP) group without lymph-nodes or distant metastasis (T_any_N_0_M_0_) (Fig. 1A). We aim to reveal special genomic aberrations according to different metastatic-potential and found early prometastastic drivers that lead to metastasis.

## Materials and methods

### Clinical samples

Primary GC tissues and matched adjacent nontumor tissues were collected from GC patients who received gastrectomy in Fudan University Shanghai Cancer Center. All patients had pathologically confirmed gastric adenocarcinoma. The clinical samples from these GC patients were kept in RNAlater at −80 °C immediately after the surgical resection. The experiments involving clinical samples from patients was approved by the Ethics Committee of Fudan University Shanghai Cancer Center and informed consent was obtained from all patients. The experiments involving animals were approved by the Ethics Committee of Fudan University Shanghai Cancer Center and the Ethics Committee of Shanghai University of Traditional Chinese Medicine.

### Whole-exome sequencing and targeted gene sequencing

The whole-exome sequencing (WES) and targeted gene sequencing (TES) were done and analyzed as described in previous studies of our team [22, 23]. TES was performed using eight selected genes, including five genes (ALK, MADCAM1, PREX2, TAF1L and TP53) with significantly higher mutation rate in HMP group than LMP group by Chi-squared test. Considering that the sample size is small and some meaningful mutations, especially low frequency mutations, could be excluded by Chi-squared test, we also included 3 genes (COL12A1, LAMA1, PRG4) with at least 1.5 times higher mutation rate in HMP group than LMP group but no statistical significance.

Genomic DNA from the frozen tissues and peripheral blood was extracted by the DNeasy Blood & Tissue Kit (Qiagen). Genomic DNA from formalin-fixed paraffin embedded (FFPE) tissues was extracted by the GeneRead DNA FFPE Kit (Qiagen). Genomic DNA libraries were prepared using the standardized protocols recommended by Illumina. Whole-exome enrichment was performed using the SeqCap EZ capture kit (Roche). Targeted gene enrichment including eight genes (TP53, COL12A1, ALK, MADCAM1, PRG4, LAMA1, PREX2 and TAF1L) was performed with the xGen Hybridization and Wash Kits (IDT). Briefly, 1 μg of DNA was sheared into short fragments (200~300 bp) using Covaris S220. The DNA fragments were then end-repaired to generate adenylated 3’ ends. Adapters with barcode sequences were then ligated to both ends of the fragments, and E-Gels were used to select DNA fragments of the targeted size. Next, ten PCR cycles were performed, and the resulting mixture was purified. After the Illumina sequencing libraries were amplified with ten PCR cycles, capture probes were added, and the mixtures were incubated for 24 h at 65 °C. The hybridized mixtures were then amplified with an additional ten PCR cycles. Captured DNA libraries were sequenced with the Illumina HiSeq 2500 Genome Analyzer, yielding 200 (2 × 100) base pairs from the final library fragments.

Sequencing reads were trimmed and filtered with Trimmomatic. The reads were aligned to the hg19 reference genome using Burrows-Wheeler Aligner, and the Genome Analysis Toolkit was used for base quality score recalibration, indel realignment and duplicate removal. VARSCAN software was used to identify somatic single-nucleotide variations and indels in WES data. VARDICT software was used to identify somatic single-nucleotide variations and indels in targeted gene sequencing data. MutsigCV software was used to analyze the significance of identified mutations with default covariate tables.

### RNA sequencing

Firstly, we extracted total RNA from GC tissues and matched normal tissues by Trizol® Reagent (Invitrogen, USA). In total, 1 μg RNA was treated with Ribo-off rRNA Depletion Kit (Vazyme) before constructing the RNA-seq libraries. VAHTS Total RNA-seq (H/M/R) Library Prep Kit for Illumina (Vazyme) were utilized to prepare RNA-seq libraries following the manufacturer’s instructions. Furthermore, ribosome depleted RNA samples (~100 ng) were fragmented and then used for first- and second-strand cDNA synthesis with random hexamer primers. The ends of the cDNA fragments were repaired by DNA End Repair Kit. Then the cDNA fragments were modified with Klenow to add an A at the 3’ end of the DNA fragments, and finally ligated to adapters. We subjected purified dsDNA to 12 cycles of PCR amplification, and sequenced the libraries by Illumina sequencing platform on a 150 bp paired-end run. Sequencing reads from RNA-seq data were aligned using the spliced read aligner HISAT2, which was supplied with the Ensembl human genome assembly (Genome Reference Consortium GRCh38) as the reference genome. Then “combat-seq” R package was used to remove batch effects. Gene expression levels were calculated by the TPM (Transcripts Per Million). We utilized the GENCODE (v25) database to get annotations of mRNA in the human genome.

### Genomic analysis

We utilized Database for Annotation, Visualization, and Integrated Discovery to perform Gene ontology (GO) analysis. The MATH score is calculated as the percentage ratio of median absolute deviation (MAD) and the median of its mutant-allele fractions at tumor-specific mutated loci, which is based on WES data of tumor and matched normal DNA: MATH = 100 × MAD/median, as described in our previous study [24]. Gene Set Enrichment Analysis (GSEA) was performed to present the gene set enriching using gene sets from Molecular Signatures Database. The scores of 64 different cell types were estimated by xCell algorithm. The relative cellular fraction of 22 immune infiltration was calculated by CIBERSORT.

### Construction of metastasis-related signature

In total, 131 genes that were not only differentially expressed between HMP tumors and matched normal tissues (*p* < 0.05), but also between M1 tumor and HMP (as well as between M1 and LMP) tumors (*p* < 0.05) were included as candidates. The prognostic signature was developed by LASSO algorithm with 200 bootstrap replications. Eleven mRNAs and long non-coding RNAs were selected for following signature. Patients in the TCGA cohort were divided into low and high riskscore groups according to the cut-off risk score (−0.885) identified by the receiver operating characteristic analysis.

### Cell lines and reagents

Human GC cell lines AGS and MGC-803 were maintained in DMEM medium (Hyclone, USA) with fetal bovine serum (10%) (BI, Israel), penicillin (100 U/ml) and streptomycin (100 µg/ml). THP-1 cells were cultured in RPMI 1640 Medium (Hyclone, USA) supplemented with 10% fetal bovine serum (BI, Israel) and 1% Penicillin-Streptomycin at 37 °C with 5% CO_2_. The cells were purchased from FuHeng Cell Center (Shanghai, China) and were authenticated by STR profiling. All cell lines were tested for mycoplasma contamination.

### Transfection

The siRNAs and the negative control were obtained from RiboBio (Guangzhou, China). Lipofectamine 2000 (Invitrogen, USA) was used to conduct transfection siRNAs and plasmids following the manufacturer’s protocol. Lipofectamine 2000 reagent and the siRNAs or plasmids were diluted in Opti-MEM medium separately. Lipofectamine 2000 reagent was incubated in room temperature for 5 min, and then mixed with diluted transfection reagent and siRNAs or plasmids. After incubating in room temperature for 20 min, the complex was added to GC cells. The media was changed 6–8 h after transfection. The transfected cells were collected for further assays after 48 h.

### Cell migration assays

The migration ability of cells was investigated by transwell assays as described previously [25, 26]. Cells were resuspended in serum free media and seeded into the chambers (Falcon, USA), while the lower chambers contained 600 μl DMEM with 20% FBS. After 24 h incubated, cells were fixed on the chambers in paraformaldehyde and stained with crystal violet. Then the upper surface was wiped off with cotton swabs and cells on the lower surface were counted under a microscope.

### Western blot assays

Western blot assays were performed as described previously [25, 26]. We lysed cells in 1 × SDS loading buffer (Beyotime, China) and incubated at 95–100 °C for 15 min. SDS-PAGE kit (EpiZyme, China) was applied to prepare the gel, and after the electrophoresis in running buffer, we transferred the separated protein lysates to polyvinylidene fluoride membrane. After being blocked in 5% skim milk for 1 h, the membrane then was incubated at 4 °C for 16 h in primary antibodies including anti-Actin (CST, #3700), anti-E-cadherin (CST, #3195), anti-Snail (CST, #3879), anti-Slug (CST, #9585), anti-Akt (CST, #4691), anti-p-mTOR(CST, #5536), anti-CCL2 (R&D Systems, #23007), anti-CD163 (Abcam, #ab87099), anti-p-Akt (CST, #4060), anti-pd-l1 (KleanAB, #P111109) and anti-MAdCAM1 (Proteintech, #21917-1-AP). Next, the membranes were washed and incubated in the secondary antibodies (CST, 1:1000) for 1 h at room temperature. Then the protein signals were visualized by ECL reagents (Millipore, USA) using the ImageQuant LAS 4000 (GE Healthcare) and quantified by ImageJ software.

### RNA extraction and quantitative real-time RT-PCR

RNA extraction and quantitative real-time RT-PCR were performed as described previously [25, 26]. Trizol® Reagent (Invitrogen, USA) was applied to extract total RNA of cells and cDNA was synthesized by using the PrimeScript RT Kit (Takara, China). The mRNA levels were measured by quantitative real-time PCR with SYBR® Premix Ex Taq™ (Takara, China). The primers were synthesis by Sangon (Shanghai, China). Each sample had three repetitions.

### Immunohistochemistry (IHC) and immunofluorescence (IF)

IHC staining were performed on paraffin sections. After antigen retrieval, paraffin sections were incubated with following monoclonal antibodies overnight at 4 °C respectively: anti-MADCAM1 (Proteintech, #21917-1-AP), anti-CD8 (Proteintech, #17335-1-AP) and anti-CD163 (Abcam, #ab87099), and followed with secondary antibody incubation. IHC staining were performed on cell. Firstly, cells were transferred onto slides. After fixed by 1% 4% paraformaldehyde, slides were incubated with primary antibodies overnight at 4 °C and followed with secondary antibody incubation.

### Co-culture assay

Cancer cells (4 × 10^5^/ml) were seeded in the upper chamber while M0 macrophages induced by THP-1 using PMA (50 ng/μl, Peprotech, USA) were seeded in the lower chamber of the 6-well transwell chambers with 0.4 um pore size polyester membrane (Corning, USA). After 48 h incubation, cancer cells or TAMs (reprogrammed-TAMs) were harvested for following assays.

### Chemotactic migration assays of T cells and macrophages

For the THP-1 and T cell chemotaxis assay, 1 × 10^5^ cells resuspended in 200 ul conditioned medium were added into the upper chamber of 5 μm pore transwell inserts (Corning, USA), while the lower chamber contained 600 ul supernatant of cancer cells or TAMs. Cells in the lower chamber were harvested and counted after 24 h incubation.

For the macrophage chemotaxis assay, THP-1 cells treated with PMA were seeded in the upper chamber with the supernatant of cancer cells or TAMs added to the lower chamber, using the 8 μm pore transwell inserts (Corning, USA). In the CCL2 neutralizing assays, the supernatant of treated cancer cells with or without CCL2 neutralizing antibody (R&D Systems, #23007, 1 ug/ml) was added into the lower chamber. Cells gone through the membrane after 48 h were fixed in 4% paraformaldehyde and stained with 0.1% crystal violet, and then quantified using five random fields.

### Human protein chemokine array

Treatepid MGC-803 (5 × 10^5^/ml) were seeded in 6-well plates for more than 48 h. Collect the supernatants of the cell cultures and complete the assay using the RayBio Chemokine Antibody Array I (RayBiotech, USA) according to the instruction manual. We firstly block the membranes at room temperature for 30 min and then incubate them in 1 ml supernatants overnight at 4 °C. After washing the membranes with the washing buffer, we sequentially incubated the membranes in 1 ml primary biotin-conjugated antibodies and 2 ml of 1000-fold diluted horseradish peroxidase-conjugated streptavidin both at room temperature for 2 h. Signals were detected using the ImageQuant LAS 4000 (GE Healthcare) and quantified by ImageJ software. Normalize the results using the positive control contained in the different membranes.

### T-cell amplification and tumor-reactive T cell killing assay

Peripheral blood mononuclear cells (PBMCs) purchased from AllCells (China) were activated with anti-CD3 antibody (100 ng/ml, Peprotech, USA), anti-CD28 antibody (100 ng/ml, Peprotech, USA) and IL-2 (10 ng/ml, Peprotech, USA). GC cell were seeded in 96-well plates and co-cultured with activated PBMCs at the ratio of 8:1 for 24 h. To detect if MADCAM1^MUT^-reprogrammed-TAM could suppress tumor-reactive T cell killing, we seeded the MGC-803 cells together with reprogrammed-TAMs (1:1) in 96-well plates. After these cells were attached, we distributed activated T-cells (MGC-803: T-cells = 1:8) into the plates and co-cultured for 24 h. The results were tested using the CCK8 kit (Dojindo, Japan) and the OD value of each well was read by BioTEK Micro-Volume Spectrophotometer (Epoch).

### PD-1 antibody treatment assay

Treated MGC-803 were seeded into 96-well plates (2 × 10^4^ cells/well), and co-cultured with activated PBMCs with or without PD-1 antibody (10 ug/ml, Innoventbio) for 24 h. To find whether reprogrammed-TAM could suppress tumor-reactive T cell killing when PD-1 was blockaded, reprogrammed-TAMs and MGC-803 cells were together seeded into the 96-well plates at the ratio of 1:5, and cell survival rates were evaluated after being co-cultured with activated PBMCs (cc:PBMCs = 1:4) and PD-1 antibody for 48 h. The results were tested using the CCK8 kit (Dojindo, Japan) and the OD value of each well was read by BioTEK Micro-Volume Spectrophotometer (Epoch).

### Subcutaneous tumor model

TAMs were reprogrammed by coculturing treated MGC-803 with M0 macrophages induced by THP-1 using PMA as above mentioned. In total, 5 × 10^6^ reprogrammed TAMs were mixed with 5 × 10^6^ MGC-803 cells, and the mixture was injected subcutaneously into the female NOD/SCID mice (6–8 weeks old, 20–25 g) were, 5 mice per group. We sacrificed these mice after 2 months and examined the lungs for metastatic loci by HE staining under microscope. Five random slices from each mouse were used for metastasis area calculation by ImageJ software.

### Tail vein metastasis model

In total, 2 × 10^6^ treated MGC-803 were injected into the tail vein of the female nude mice (6–8 weeks old, 20–25 g), five mice per group. After 2 months, we sacrificed all mice and examined the lungs for metastatic loci by HE staining under microscope. Five random slices from each mouse were used for metastasis area calculation by ImageJ software.

### Statistical analysis

No statistical methods were used to determine the sample size. No randomization was used and the investigators were not blinded to allocation during experiments. Statistical analysis was performed using Pearson chi-square test, Mann–Whitney *U* test, Log-rank test, Kruskal–Wallis test, pairwise *t-*test, Student’ *t* test and Pearson correlation test, using R version 3.5.1 and GraphPad Prism Software 7.0. The *p* value < 0.05 was set as the statistical significance.

## Results

### Overview of WES

We performed WES on tumor and paired normal DNA from 192 Chinese GC patients (Fig. 1B). Demographics and clinicopathologic characteristics of our cohort was shown in the Supplementary Table 1. In total, 38,641 non-synonymous somatic mutations in 13,191 genes were identified (Fig. 1C). Mutational signatures analysis based on 96 possible somatic substitutions showed that a signature associated with defective DNA mismatch repair is one of the dominant signatures (Fig. 1D). The median number of mutations was 40 per sample.

### Comparison of cancer driver mutations among populations

To compare the mutational pattern of FUSCC cohort in this study with other populations, we obtained the TCGA GC cohort (*n* = 395). The median number of mutations in TCGA cohort was 107/sample, which is significantly higher than FUSCC cohort. We include 30 genes described as “cancer driver mutations” in previous literatures and almost all of them had higher mutation rate in TCGA cohort (28/30) (Supplementary Table 2). This suggests that Chinese GC patients show different characteristics from TCGA cohort and may have different drivers, which were worth exploring.

### The relationship between mutation of DNA damage and repair (DDR) related genes and mutational load

We next examined the relationship between mutation status of 35 key DDR related genes and the number of mutations (Supplementary Table 3). Tumors with mutation of any MMR genes have obviously higher mutational load than others (Median: 953.5 vs. 36.5). To reduce the influence of MMR genes status on mutational load, the number of mutations according to DDR genes were calculated in tumors with wild-type MMR genes. We found that mutation status of these DDR genes was all associated with higher mutational load.

### Mutational signatures and intra-tumor heterogeneity (ITH) according to metastatic-potential

**Fig. 2:**
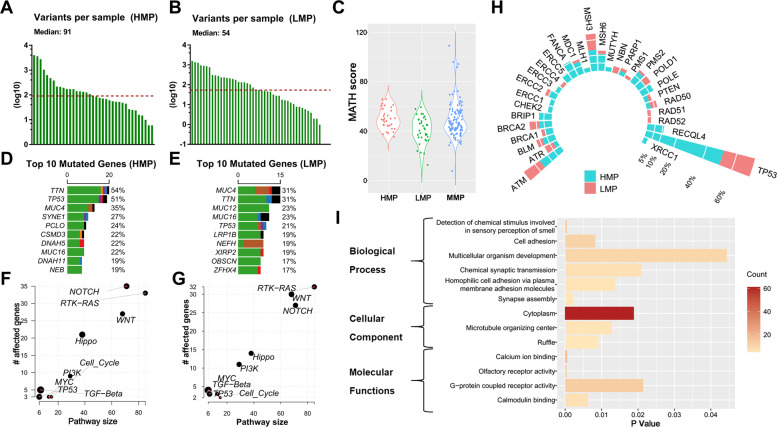
Alterations landscape according to metastatic-potential. Numbers of variants per sample in HMP group (**A**) and LMP group (**B**). **C** Intra-tumor genetic heterogeneity measured by MATH (Mutant-allele tumor heterogeneity). Top 10 mutated genes and their mutation rate in HMP group (**D**) and LMP group (**E**). Enrichment of known Oncogenic Signaling Pathways in HMP group (**F**) and LMP group (**G**). Each gene is represented as a dot, while their horizontal and vertical coordinates indicate numbers of genes in pathways and numbers of mutated genes in pathways, respectively. Sizes of dots represent the proportion of mutated genes in pathways. **H** The mutational rates of 35 genes known as DNA damage and repair genes in HMP group and LMP group. **I** Functional analysis of Gene Ontology (GO) analyzed the annotation of 176 candidate driver mutations.

To explore the genomic aberrations according to metastatic-potential, we separated samples into groups based on metastatic-potential. Besides HMP (*N* = 37) and LMP group (*N* = 48), all the rest of samples (T_2-4_N_+_M_0_, *N* = 107) were classified as middle metastatic-potential (MMP) group. The most frequent transition in HMP group was C > T, and C > A was more common in LMP group (Supplementary Fig. 1). Median number of mutations was significantly higher in HMP group, compared to LMP group (91 vs. 54) (Fig. 2A, B). Intra-tumor genetic heterogeneity measured by mutant-allele tumor heterogeneity (MATH) was higher in HMP group (mean value = 49.08) than LMP group (mean value = 44, *p* < 0.001) (Fig. 2C).

### Identification of significant mutations according to metastatic-potential

We next identified potential prometastatic mutational genes. The top mutated genes in two groups were shown in Fig. 2D, E. Mutational genes showed enrichments for known oncogenic pathways, while NOTCH and RTK-RAS pathways are tops in HMP and LMP groups respectively (Fig. 2F, G). Interestingly, we analysis some known DDR related genes and found that the mutational rate of most (24/31) were higher in HMP group (Fig. 2H).

We identified 176 genes with significantly higher mutation rate in HMP group (>10%) than LMP group (*p* < 0.05) (Supplementary Table 4). All these genes had lower mutational rates in MMP group than HMP group, which illustrates the reliability of our grouping method. GO analysis annotated these mutations and the top-ranking biological process included “Cell Adhesion” (*p* = 0.008) (Fig. 2I).

### Further validation of candidate metastasis-related genes in vitro

We then explore the roles of candidate metastasis-related genes in metastasis by in vitro experiments. We found that knockdown of RB1CC1, DLL1, PRG4, PCLO, and NBPF10 increased, while knockdown of TAF1L, PREX2 and ATXN3 and upregulation of TP53 and MADCAM1 and KRTAP5-5 impaired the migration ability of GC cells, which suggested their potential roles in metastasis of GC (Supplementary Fig. 2).

### Candidate prometastatic mutations associated with distant metastasis by TES

**Fig. 3:**
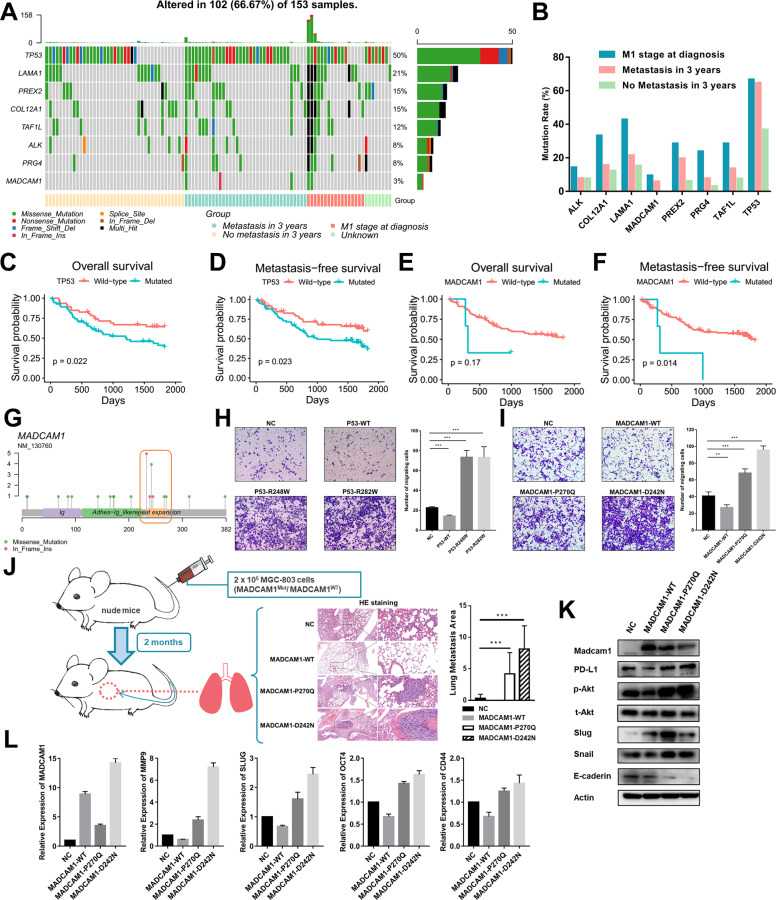
Targeted-exome sequencing (TES) revealed that mutation of MADCAM1 and TP53 promote metastasis. **A** Landscape of genetic alterations from TES of 153 FUSCC GC patients. **B** The mutational rate of genes in patients with distant metastasis at diagnosis, patients with metastasis within 3 years after surgery, and patients with no metastasis within 3 years after surgery. The impact of mutations in TP53 on overall survival (**C**) and metastasis-free survival analysis (**D**). The impact of mutations in MADCAM1 on overall survival (**E**) and metastasis-free survival analysis (**F**). **G** Mutational distribution on MADCAM1 from WES and TES samples. **H** The migration ability of AGS was significantly inhibited when transfected a vector expressing wild-type P53, and enhanced when transfected vectors expressing P53 R248W or R282W mutants. **I** The migration ability of AGS was significantly inhibited when transfected a vector expressing wild-type MADCAM1, and enhanced when transfected vectors expressing MADCAM1 P270Q or D242N mutants. **J** 2 × 10^6^ treated MGC-803 were injected into the tail vein of nude mice (5 mice/group). MGC-803 transfected MADCAM1^P270Q^ or MADCAM1^D242N^ vector generated increased lung metastases burden. The representative figures of HE staining were shown. **K** The effect of wild-type and mutated MADCAM1 on the EMT markers and PD-L1 in Western blot assay. **L** The effect of wild-type and mutated MADCAM1 on the EMT and stem cell markers in qPCR assay. Bars, ±SD; *p < 0.05; **p < 0.01. ***p < 0.001.

We further performed TES on another cohort of 153 GC patients using eight candidate genes with higher mutation rate in HMP group (Fig. 3A and Supplementary Table 5). All of these eight genes mutated more frequently in patients with distant metastasis at diagnosis, especially than those did not have metastasis within 3 years after surgery. In patients with metastasis within 3 years after surgery, the mutational rates of TP53, MADCAM1, PREX2, PRG4 and TAF1L were nearly twice or more than patients without metastasis and statistical significances were observed in TP53 and MADCAM1 (Fig. 3B). Though with low mutational rate, MADCAM1 mutations were only in patients with metastasis within 3 years after surgery (5.88%) and at diagnosis (9.52%). Consistent with their top ranking in MutsigCV analysis, mutation status of TP53 and MADCAM1 were significantly associated with worse metastasis-free survival (MFS) (Fig. 3C–F). Mutation status of TP53 and MADCAM1 were still independent risk factors for MFS after adjusting possible confounding factors (Supplementary Table 6). Mutation hotspots from WES and TES samples at the protein encoded by MADCAM1 were identified in “Repeat Expansion” region between amino acids 227 and 271 (Fig. 3G). TP53 and MADCAM1 hotspot mutations could enhance migration abilities of GC cells (Fig. 3H, I and Supplementary Fig. 2C, D). The roles of MADCAM1 and its mutants in proliferation ability of GC cells were confirmed by in vitro assays. (Supplementary Fig. 2E, F). We further observed increased lung metastasis generated by MADCAM1^P270Q^ or MADCAM1^D242N^ injected into the tail veins of nude mice (Fig. 3J). The expression of some EMT markers (Slug, Snail, E-cadherin), stem-like cell markers (Oct4, Cd44) were up-regulated in AGS transfected with vectors expressing MADCAM1^P270Q^ or MADCAM1^D242N^, compared with MADCAM1^WT^ (Fig. 3K, L).

### Transcriptional landscape and tumor molecular heterogeneity according to metastatic-potential

**Fig. 4:**
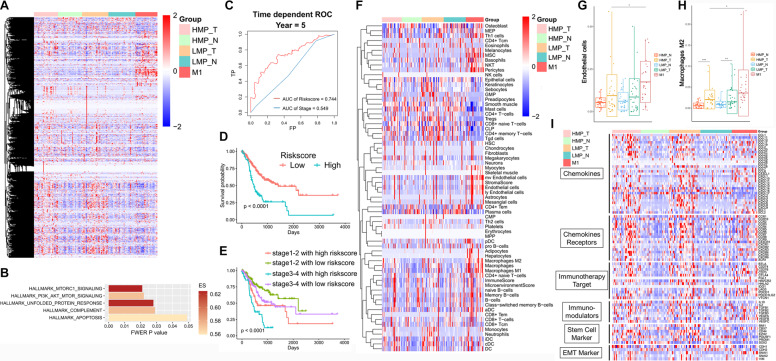
Transcriptional pattern according to metastatic-potential. **A** Heatmaps analysis on RNA expression of tumor and paired normal tissues from 87 Chinese GC patients. Samples are in columns, grouped by metastatic-potential and sample type (tumor or normal tissues). **B** Gene set enrichment analysis (GSEA) of gene signatures enrichment in HMP tumors vs. HMP normal tissues, but no enrichment in LMP tumors vs. LMP normal tissues. **C**–**E** Prognostic value and accuracy of the the integrated mRNA-lncRNA signature and traditional TNM stage in TCGA cohort. **F** The scores of 64 different cell types by xCell algorithm. **G**, **H** The scores of endothelial cells and M2 macrophage in different group. **I** Heatmaps analysis on mRNA expression for six types of immune-related genes: cytokines, cytokine receptors, immunotherapy targets, immunomodulators, stem cell markers, EMT markers.

We then performed analysis on transcriptomes of tumor and paired normal tissues from 87 GC patients, including 29 HMP patients, 32 LMP patients and 26 patients with distant metastasis at diagnosis (M1). Hierarchical clustering method could distinguish tumors from normal tissues and also HMP from LMP tumors (Fig. 4A). GSEA demonstrated that HMP group was enriched with pathways including mTORC1 and PI3K/AKT/mTOR signaling (Fig. 4B). The molecular heterogeneity was then demonstrated by the number of differentially expressed genes (DEGs) under certain cut-off *p* value between tumor and matched normal tissues [27]. As expected, the number of DEGs in HMP group was more than LMP group under different *p* value or adjusted *p* value, indicating higher heterogeneity in HMP group, which is consistent with above results of ITH calculated by MATH (Supplementary Table 7).

### Construction of metastasis-related signature to predict prognosis

In total, 131 genes with high expression in HMP and M1 tumors were included as candidates for prognostic signature by LASSO algorithm in TCGA cohort. A signature consisting of 11 mRNAs and long non-coding RNAs were derived and the integrated mRNA-lncRNA signature had better prognostic value (area under curve (AUC) = 0.744) than traditional TNM stage (AUC = 0.549) (Fig. 4C). Patients with high riskscore had significantly poor survival (Fig. 4D). We also find that the survival of patients with same TNM stage could be distinguished by the riskscore (Supplementary Fig. 3A). In addition, patients with stage III-IV and low riskcore, have better survival than patients with stage I-II and high riskcore, which also demonstrated the superior of the signature than traditional TNM stage (Fig. 4E). In FUSCC cohort, we observed more proportion of M1 (50.00% vs. 9.30%) and less LMP (20.45% vs. 53.49%) in high riskcore than low riskcore group (Supplementary Fig. 3B).

### Enhanced suppressive immune phenotype in HMP patients

We analyzed the level of immune cell infiltration by 66 markers of different immune cell types according to GSVA scores (Supplementary Fig. 3C) [28]. The tumors of HMP group exhibited remarkably enhanced immune cell infiltration than normal tissues (*p* = 0.0193), but there was no significant difference in LMP group.

We further investigated the scores of 64 different cell types by xCell algorithm (Fig. 4F). In tumors of HMP group, we observed elevated endothelial cells and M2 macrophage than normal tissues of HMP group or tumor tissues of LMP group (Fig. 4G, H).

Additionally, some EMT markers (i.e., SLUG), stem-like cell markers (i.e., EZH2), chemokines (i.e., CCL11, CCL13, CXCL6), chemokine receptors (i.e., CCR6, CCR7, CCR9, CCR10), immune checkpoint targets (i.e., CD70, IDO1) and other immunomodulators (i.e., TGFB1, VEGFA, VEGFC) were only significantly upregulated in HMP tumors but not in LMP group (Fig. 4I).

### MADCAM1 mutants associated with chemotactic migration of T cells and macrophages

**Fig. 5:**
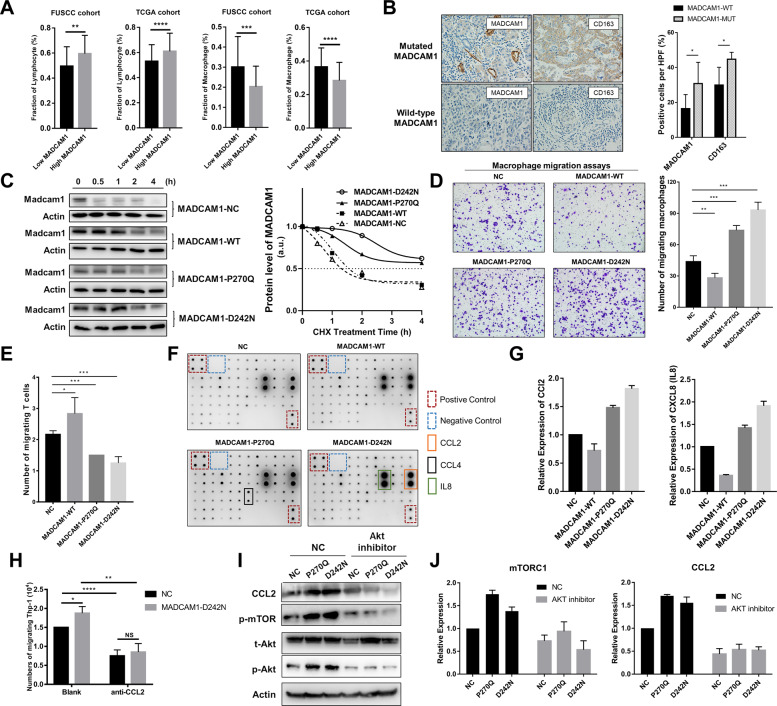
MADCAM1 mutants associates with chemotactic migration of T cells and M2 macrophages. **A** The expression of MADCAM1 show a significant positive correlation with the fraction of lymphocyte and a negative correlation with the fraction of macrophages, in both TCGA cohort and FUSCC cohort. **B** Immunohistochemistry (IHC) analysis showed that the pericellular staining intensity of MADCAM1 in the tumor cells of samples carrying mutated MADCAM1 was significantly stronger than those carrying wild-type MADCAM1. Additionally, CD163 was significantly positively stained in the infiltrating immune cells of samples with mutated MADCAM1, when compared with those with wild-type MADCAM1. **C** Effect of MADCAM1^P270Q^ and MADCAM^D242N^ on stability of the Madcam1 protein. GC cells transfected vectors expressing wild-type MADCAM1 (MADCAM1-WT), MADCAM1^P270Q^ mutants (MADCAM1-P270Q), MADCAM1^D242N^ mutants (MADCAM1-D242N) or negative control (MADCAM1-NC) were treated with 100 μg/ml cycloheximide (CHX) and then were harvested for western blot at indicated time points. Western blot data are quantified by ImageJ software. **D** Macrophage migration assay by the supernatants from MADCAM1^WT^, MADCAM1^MUT^ and control GC cells. **E** T cell migration assay by the supernatants from MADCAM1^WT^, MADCAM1^MUT^ and control GC cells. **F** The chemokine profiles of corresponding supernatants detected by Human Chemokine Antibody Arrays. **G** Expression of CCL2 and IL8 in supernatant from MADCAM1^WT^, MADCAM1^MUT^ and control GC cells. **H** Blockading CCL2 by anti-CCL2 antibody reduced migration of macrophages, especially in MADCAM1^D242N^ GC cells. **I** The p-Akt, CCL2 and p-mTOR was blocked by Akt inhibitor Perifosine, while the total levels of Akt were hardly changed. **J** The expression of p-mTOR and CCL2 was obviously suppressed after inhibition of Akt in MADCAM1^P270Q^ and MADCAM1^D242N^ GC cells. Bars, ±SD; *p < 0.05; **p < 0.01. ***p < 0.001.

Considering the roles of MADCAM1 mutants in metastasis, we next examined if MADCAM1 was involved in immune infiltration and immune escape. The expression of MADCAM1 show a significant positive correlation with lymphocyte and a negative correlation with macrophages calculated by CIBERSORT, in both TCGA and FUSCC cohort (Fig. 5A and Supplementary Fig. 4A, B). The tumors carrying mutational MADCAM1 had more macrophage infiltration than others in TCGA cohort (Supplementary Fig. 4C). We also observed that the expression of MADCAM1 significantly associated with many chemokines, chemokine receptors and immunomodulators, including CD274 that coding PD-L1 (Supplementary Table 8). IHC assays showed that M2 macrophage marker CD163 in infiltrating immune cells and MADCAM1 in the tumor cells was significantly positively stained in GC samples carrying mutated MADCAM1 (Fig. 5B). So, we investigated whether mutated MADCAM1 lead to alteration in stability of Madcam1 protein. We examined the level of Madcam1 protein in the presence of 100 μg/ml cycloheximide, a protein-synthesis inhibitor. The half-life time of Madcam1 protein for MADCAM1^WT^ cells was ~1.5 h, whereas over 4 h in MADCAM1^P270Q^ and MADCAM1^D242N^ cells (Fig. 5C).

We next explored if MADCAM1 mutants affects chemotactic migration of T cells and M2 macrophages. The supernatants from MADCAM1^WT^ GC cells induced migration of activated T cells, and reduced migration of M2 macrophages. Meanwhile, the supernatants from MADCAM1^P270Q^ and MADCAM1^D242N^ GC cells reduced migration of T cells, and induced migration of M2 macrophages (Fig. 5D, E).

### MADCAM1 mutants recruited macrophages by regulating CCL2 through Akt/mTOR axis

Chemokine profiles detected by Human Chemokine Antibody Arrays presented higher CCL2 and IL8 in supernatants of MADCAM1^D242N^ GC cells compared with that of MADCAM1^WT^ GC cells (Fig. 5F, G). Further, blocking CCL2 reduced migration of macrophages, especially in MADCAM1^D242N^ GC cells (Fig. 5H), which demonstrated that the ability of MADCAM1^D242N^ in recruiting macrophages was partly dependent on CCL2. In western blot assay (Fig. 3K), phosphorylation levels of Akt were obviously enhanced in MADCAM1^P270Q^ and MADCAM1^D242N^ GC cells, while the total levels were hardly changed. The expression of p-mTOR and CCL2 was suppressed after inhibiting Akt in MADCAM1^P270Q^ and MADCAM1^D242N^ GC cells (Fig. 5I, J). These results indicated that MADCAM1 mutants promotes recruitment of macrophages by regulating CCL2 through Akt/mTOR axis.

### MADCAM1 mutations promoted PD-L1-mediated immune escape

**Fig. 6:**
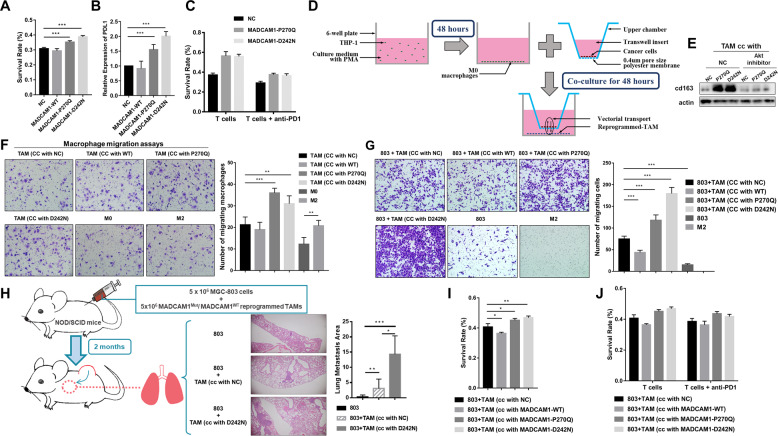
MADCAM1 mutations are involved in immunosuppression. **A** The survival rate of MADCAM1^WT^, MADCAM1^MUT^ and control GC cells after co-culture with activated PBMC. **B** The relative expression of CD274 (PD-L1) in MADCAM1^WT^, MADCAM1^MUT^ and control GC cells. **C** The survival rate of MADCAM1^WT^, MADCAM1^MUT^ and control GC cells after co-culture with activated PBMC in the absence or presence of anti-PD-1 antibodies. **D** Diagrammatic representation of reprogramming macrophage by co-culture of GC cell with PMA treated THP-1. **E** Strong CD163 by western blot were observed in MADCAM1^P270Q^-reprogrammed TAMs and MADCAM1^D242N^-reprogrammed-TAMs. **F** Migration assays of THP1 macrophage by the supernatants from corresponding reprogrammed TAMs, M0 or M2 macrophages. **G** The migration assays of AGS in the absence or presence of corresponding reprogrammed TAMs. **H** The NOD/SCID mice (5 mice/group) co-injected subcutaneously with MGC-803 cells and blank control or MADCAM1^WT^-reprogrammed TAM or MADCAM1^D242N^ -reprogrammed TAM. After 2 months, the mice co-injected with MADCAM1^D242N^ -reprogrammed TAM and MGC-803 cells have more area of lung metastatic loci. **I** The survival rate of GC cells after co-culture with activated PBMC along with corresponding reprogrammed TAMs. **J** The survival rate of GC cells after co-culture with activated PBMC along with corresponding reprogrammed TAMs in the absence or presence of anti-PD-1 antibodies. Bars, ±SD; *p < 0.05; **p < 0.01. ***p < 0.001.

T-cell-mediated killing assays were performed to determine if MADCAM1 mutations affects T cell cytotoxic activity. MADCAM1^P270Q^ and MADCAM1^D242N^ GC cells presented significantly higher survival rate than MADCAM1^WT^ GC cells and control after co-culture with activated PBMC (Fig. 6A). Consistent with above correlation analysis, up-regulated CD274 (PD-L1) expression was confirmed in MADCAM1^MUT^ GC cells by qPCR (Fig. 6B) and Western blot assays (Fig. 3K). Further, blocking PD-1 by anti-PD-1 antibody could facilitate T-cell-mediated killing in MADCAM1^MUT^ GC cells (Fig. 6C). These results indicated that MADCAM1^P270Q^ and MADCAM1^D242N^ mutations could upregulated PD-L1 and suppress PD-L1-mediated T cell killing.

### MADCAM1 mutations induced M2-like polarization of macrophages by CCL2 through Akt /mTOR axis

We then explore the role of MADCAM1 mutations in macrophage polarization by co-culture of GC cell with PMA treated THP-1 (Fig. 6D). The expression of M2 markers, including ARG1, IL10 and CD163 was higher in MADCAM1^P270Q^-reprogrammed tumor-associated macrophages (TAMs) and MADCAM1^D242N^-reprogrammed-TAMs, but lower or same in MADCAM1^WT^-reprogrammed-TAMs compared with control (Supplementary Fig. 5A). Strong CD163 immunofluorescence staining and M2-like morphology were observed in MADCAM1^MUT^-reprogrammed-TAMs (Supplementary Fig. 5B). M2 markers and MTOR were up-regulated in reprogrammed-TAMs, which could be reversed by Akt inhibitor. (Fig. 6E and Supplementary Fig. 5C). Blocking CCL2 could reversed the increased M2 markers in MADCAM1^D242N^ reprogrammed-TAM (Supplementary Fig. 5D).

### MADCAM1^MUT^-reprogrammed-TAMs attracted more macrophage and promote migration of GC cells

We next test if MADCAM1^MUT^-reprogrammed-TAMs promote migration of macrophages and GC cells. Macrophage migration assays were performed by supernatants from reprogrammed-TAMs. MADCAM1^P270Q^-reprogrammed and MADCAM1^D242N^-reprogrammed-TAMs significantly promote migration of macrophages (Fig. 6F). The GC cells co-cultured with MADCAM1^MUT^-reprogrammed-TAMs showed increased migration ability compared with MADCAM1^WT^-reprogrammed-TAMs, which could be reversed by Akt inhibitor (Fig. 6G and Supplementary Fig. 5E). We next validated these results in vivo. MADCAM1^MUT^-reprogrammed or MADCAM1^WT^-reprogrammed-TAMs were mixed with MGC-803 cells and co-injected subcutaneously into NOD/SCID mice and MADCAM1^D242N^-reprogrammed TAM group have more lung metastatic loci (Fig. 6H).

### MADCAM1^MUT^-reprogrammed-TAMs suppressed tumor-reactive T cell killing which cannot be reversed by anti-PD-1 antibody

To determine whether MADCAM1^MUT^-reprogrammed-TAMs play role in immune escape, T-cell-mediated killing assays were performed in the presence of reprogrammed-TAMs. MADCAM1^P270Q^-reprogrammed and MADCAM1^D242N^-reprogrammed-TAMs could protect GC cells from tumor-reactive T cells killing (Fig. 6I). The expression of CD274, as well as some EMT markers (SLUG, MMP9), OCT4 and chemokines (IL4, IL13), were up-regulate in GC cells co-cultured with MADCAM1^P270Q^-reprogrammed or MADCAM1^D242N^-reprogrammed-TAMs, compared with GC cell co-cultured with MADCAM1^WT^-reprogrammed-TAMs (Supplementary Fig. 5F). However, blocking PD-1 by anti-PD-1 antibody could hardly facilitate T-cell-mediated killing in GC cell co-cultured with MADCAM1^MUT^-reprogrammed-TAMs (Fig. 6J). These results proved that MADCAM1^MUT^-reprogrammed-TAMs could contribute to tumor development by protecting GC cells from tumor-reactive T cells killing, which doesn’t depend on PD-L1.

## Discussion

Identification of driver molecular alterations is critical for cancer research and development of new target drugs and treatment strategies. Traditional comparative analysis between primary and metastasis tumors or among metastases could miss out prometastatic drivers, since it is hard to distinguish them from protumorigenic or passenger mutations. Here, we tried a novel strategy to identify early drivers in primary gastric tumors. Integrated DNA and RNA sequencing and comparative analysis according to different metastatic-potential revealed some prometastatic drivers and immune suppressive microenvironment established in EGC. It provides evidence for early metastatic dissemination in GC, which have already been quantitatively validated in CRC by Hu et al. [29]. WES demonstrated distinct genomic pattern and higher mutation load in HMP groups. We revealed higher mutational rate of MMR and some DDR genes, which might illustrate this finding, since mutations in DDR gene could drive genomic instability and then generate more somatic mutations, including prometastatic drivers [30, 31]. Higher mutation load and heterogeneity in HMP group indicated that multiple molecular alteration involved in metastasis.

Further, we identified 176 candidate prometastatic mutations and mutation of TP53, MADCAM1 and KRTAP5-5 enhanced migration of GC cells, which suggested the potential prometastatic roles of these candidate mutations. We then included 8 genes in further TES. Among them, mutation status of TP53 and MADCAM1 was independent risk factors for MFS. Furtherly, in vitro experiments indicated that TP53 and MADCAM1 mutants directly promoted migration of GC cells. The results indicated that our strategy to identify early prometastatic drivers was credible and challenged traditional opinions that there were few driver mutations involved in metastasis [3, 4, 32].

Some mutations identified in this study was previously found to involve in development and metastasis of cancer. TP53 mutant could gain additional functions to promote growth and metastatic-potential of tumor cells in many types of cancer [10, 33, 34]. Frequent PREX2 and PRG4 mutations was observed in melanoma and mutant PREX2 accelerated tumor formation [35]. As a member of the immunoglobulin family involving in lymphocyte adhesion, the roles of MADCAM1 mutants in tumor metastasis were firstly reported.

To investigate the association between metastatic-potential and gene expression on RNA level, we performed RNA sequencing. Higher heterogeneity measured by the number of DEGs was observed in HMP group, which was consistent with MATH algorithm. An integrated mRNA-lncRNA signature based on DEGs in HMP group was constructed and its prognostic value was better than traditional TNM stage. The combination of signature and stage could help guiding prognosis prediction and clinical treatment decision.

Upregulation of some EMT and stem-like cell markers not only confirmed that HMP group had HMP, but also indicated metastasis-related mutations might promote metastasis through EMT and stemness mechanisms. Mutated TP53 was previously reported to promote EMT and metastatic ability of cancer cells. Here, we demonstrated that mutated MADCAM1 also involved in process of EMT and metastasis of GC cells. Our previous studies found that some stem-like cell markers (i.e., CD44) could enhancing EMT and metastatic ability of cancer cells [25, 26, 36, 37].

Based on the finding that upregulation of immune cell infiltration in HMP group, we further investigated that if immune escape mechanism involved in early metastasis of GC. Tumor immune microenvironment was reported to play a critical role in cancer metastasis [32, 38, 39]. The infiltration of activated neutrophil, TAM, and Treg cells played roles in progression of dysplastic mucosa in early stage of gastrointestinal neoplasia [40–42]. We provided a comprehensive analysis of immune microenvironment in EGC for the first time and found immune suppressive microenvironment was presented in HMP group.

Does the immune suppressive microenvironment of HMP group result from mutations and finally contribute to cancer cells metastasis? Previous articles reported that some driver mutations participate in remodeling immune microenvironment, for example, TP53 mutant were reported to be involved in TAMs reprogramming to promote metastasis in colon cancer [43]. The hotspot mutational sites observed by us in TP53 and MADCAM1 indicated their potential gain-of-function (GOF) effects. We demonstrated that MADCAM1-mutated proteins act as GOF mutants and lead to alteration in stability of the Madcam1 protein. The roles of MADCAM1 mutants in tumor metastasis is a novel interesting funding. MADCAM1 was reported to be highly expressed in gastrointestinal mucosal endothelium and plays a role in leukocyte traffic into the mucosal immune compartment [44–47] and the roles of MADCAM1 mutants in tumor metastasis had never been reported before. Here, we found that MADCAM1 mutants could not only directly promote tumor metastasis, but also could trigger tumor metastasis by establishing complicated immunosuppressive microenvironment. On the one hand, MADCAM1 mutants reduced chemotactic migration of T cells by regulating chemokines and promotes PD-L1-mediated immune escape. On the other hand, MADCAM1 mutants induced chemotactic migration and M2-like polarization of macrophages by regulating CCL2 through Akt /mTOR axis. MADCAM1^MUT^-reprogrammed-TAMs attracted more macrophages and promoted migration of GC cells in vivo and in vitro. Additionally, MADCAM1^MUT^-reprogrammed-TAMs could suppress tumor-reactive T cell killing to help immune escape, which couldn’t be reversed by anti-PD-1 antibody. It indicated that MADCAM1^MUT^-reprogrammed-TAMs and Akt/mTOR signaling pathway may contribute to resistance to anti-PD-1/PD-L1 therapy. Further in vivo work is warranted to verified the role of CCL2 and Akt/mTOR axis in MADCAM1^MUT^-reprogrammed immune microenvironment in GC. Regardless of it, our results demonstrate the potential clinical value of further work exploring potential therapies that target MADCAM1 mutations in GC.

**Fig. 7:**
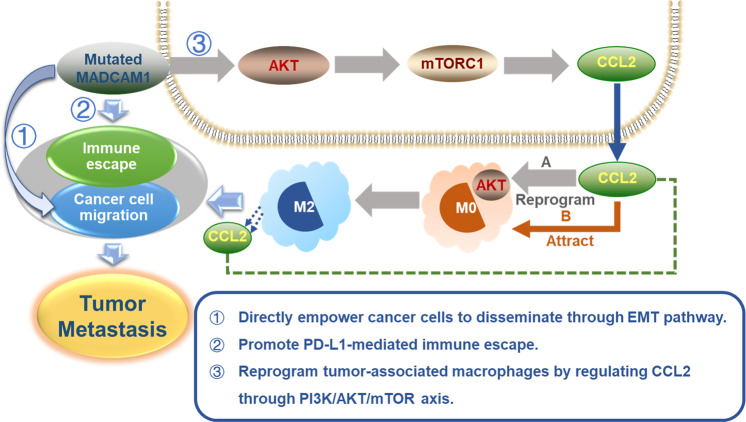
Proposed model of how early drivers (including MADCAM1) involved in tumor metastasis. Mutated MADCAM1 could drive tumor metastasis by directly empowering cancer cells to disseminate or establishing an immunosuppressive microenvironment.

In conclusion, our results showed that the GCs with different metastatic-potential are distinguishable at the genetic level. A number of potential metastatic driver mutations was revealed in this study including known cancer-associated genes TP53. We also discovered some novel driver mutations, such as MADCAM1. The results proved our hypothesis that driver mutations in early-onset metastatic GC could drive metastasis not only by directly empowering cancer cells to disseminate but also by establishing an immunosuppressive microenvironment. Driver mutations and the remodeling of the immune microenvironment simultaneously promoted the metastasis and development of tumor. The summary diagram was shown in Fig. 7. Here we demonstrated that despite a low mutational rate in GC, GOF MADCAM1 mutations could play driver roles in promoting tumor metastasis through establishing immunosuppressive microenvironment, and MADCAM1^MUT^-reprogrammed-TAMs and Akt/mTOR signaling pathway may contribute to resistance to anti-PD-1/PD-L1 therapy. Previous articles reported that TP53 mutations could reprogram TAMs to promote tumor progression and metastasis. These findings indicated that for GC patients carrying mutated MADCAM1 or TP53, combination of anti-PD-1/PD-L1 therapy and inhibition of TAMs would be a choice. The upregulation of checkpoint targets in HMP tumors also provides a theoretical basis for the early intervention of immune checkpoint inhibitors in EGC with high risk of metastasis in the future. This study increased the understanding of molecular landscape of GC patients and provided possibility for future target therapy.

## Supplementary information

aj-checklist

Supplementary Figures 6

Supplementary Figures 1-5

Supplementary Tables 1-8

## Data Availability

The data of the current study available from the corresponding author on reasonable request.
