# Supplementary figures and images for: Integrated DNA and RNA sequencing reveals early drivers involved in metastasis of gastric cancer
Source: Cell Death Dis. 2022 Apr 21;13(4):392. doi: 10.1038/s41419-022-04838-1 (PMC9023472; doi:10.1038/s41419-022-04838-1)

**Supplementary Figures 6. Uncropped original western blots images used in the article.**

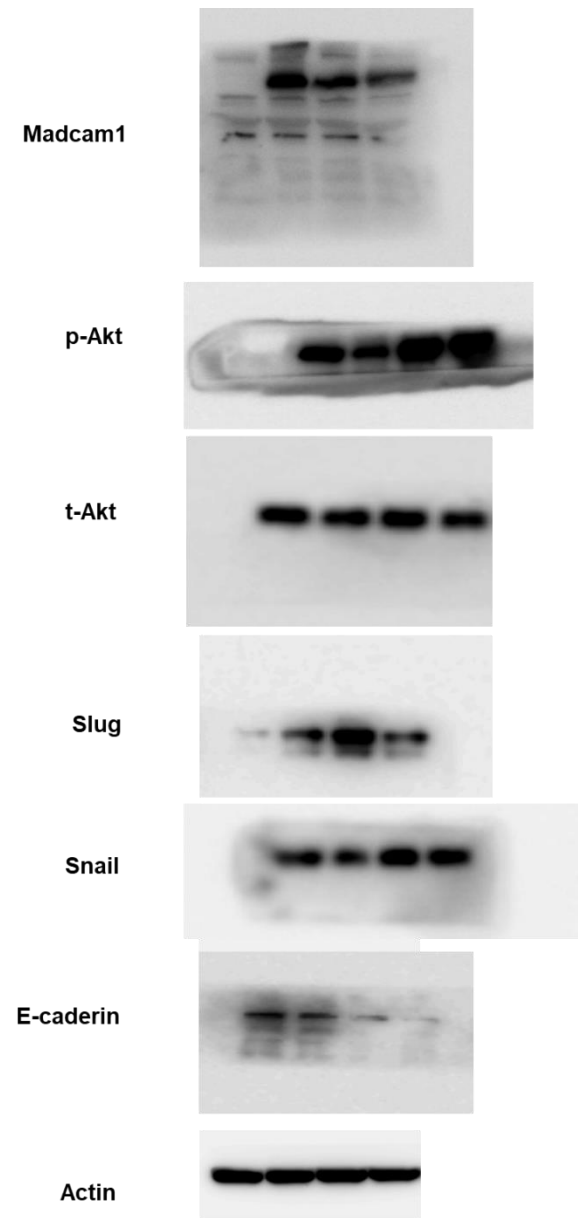

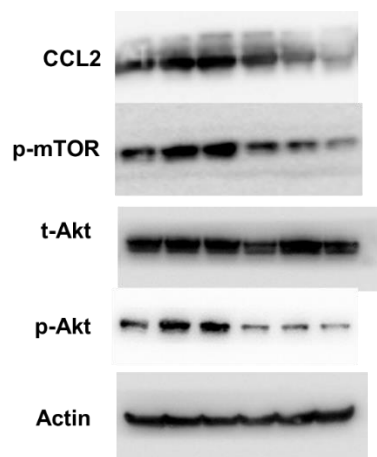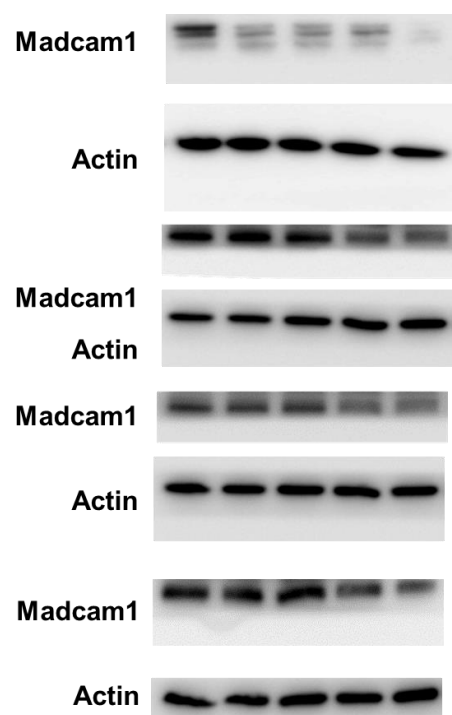

Supplement: Supplementary file 2 — Supplementary Figures 6 [file 41419_2022_4838_MOESM2_ESM.pdf]
