# Supplementary material for: Integrated DNA and RNA sequencing reveals early drivers involved in metastasis of gastric cancer
Source: Cell Death Dis. 2022 Apr 21;13(4):392. doi: 10.1038/s41419-022-04838-1 (PMC9023472; doi:10.1038/s41419-022-04838-1)

Legends of Supplementary Figures

Supplementary Figure 1. Summary of variants in HMP and LMP group.

(A, B) Summary of variants in HMP (A) and LMP group (B), including variant classification, variant type (SNP/INS/DEL) and SNV class.

Supplementary Figure 2. The roles of candidate metastasis-related genes in migration of GC cells

(A) Knockdown of RB1CC1, DLL1, PRG4, PCLO, and NBPF10 increased, while knockdown of TAF1L, PREX2 and ATXN3 impaired the migration ability of AGS ($P<0.05$).

(B) Upregulation of KRTAP5-5^{WT} increased the migration ability of AGS, while upregulation of KRTAP5-5^{A53_C62del} further increased the migration ability of AGS more.

(C) Upregulation of TP53^{WT} impaired the migration ability of MGC-803, while upregulation of TP53^{R248W} and TP53^{R282W}increased the migration ability of MGC-803.

(D) Upregulation of MADCAM1^{WT} impaired the migration ability of MGC-803, while upregulation of MADCAM1^{P270Q} and MADCAM1^{D242N} increased the migration ability of MGC-803.

(E) CCK8 assays demonstrated that upregulation of MADCAM1^{WT} impaired the proliferation ability of MGC-803, while upregulation of MADCAM1^{P270Q} and MADCAM1^{D242N} increased the proliferation ability of MGC-803.

(F) Clone formation experiments demonstrated that upregulation of MADCAM1^{WT} impaired the proliferation ability of MGC-803, while upregulation of MADCAM1^{P270Q} and MADCAM1^{D242N} increased the proliferation ability of MGC-803.

Supplementary Figure 3. Transcriptional landscape: Construction of metastasis-related signature to predict prognosis and analysis of immune cell infiltration.

(A) The survival of patients with same TNM stage according to riskscore by signature.

(B) The proportion of HMP, LMP, and M1 group in high riskcore and low riskcore group.

(C) The level of immune cell infiltration by a list of 66 immune markers containing cell surface markers of different immune cell types.

Supplementary Figure 4. MADCAM1 associates with macrophages in TCGA cohort.

(A, B) The expression of MADCAM1 show a significant negative correlation with the fraction of macrophages and a significant positive correlation with the fraction of lymphocyte.

(C) The tumors carrying mutational MADCAM1 had more fraction of macrophage infiltration than others.

Supplementary Figure 5. The expression of some markers in reprogrammed TAMs and GC cell co-cultured with reprogrammed-TAMs.

(A) The expression of M2 markers, including ARG1, IL10 and CD163 was higher in $MADCAM1^{P270Q}$-reprogrammed tumor-associated macrophages (TAMs) and $MADCAM1^{D242N}$-reprogrammed-TAMs, but lower or same in $MADCAM1^{WT}$-reprogrammed-TAMs compared with the control.

(B) Immunofluorescence staining of CD163 and morphology observed in $MADCAM1^{WT}$, $MADCAM1^{MUT}$ and control reprogrammed TAMs. The mean intensity of CD163 was

normalized to nuclear DAPI staining and was quantified by ImageJ software.

(C) The expression of M2 markers, including ARG1 and CD163 was higher in MADCAM1^{P270Q}-reprogrammed TAMs and MADCAM1^{D242N}-reprogrammed-TAMs, which could be reversed by AKT inhibitor.

(D) Blockading CCL2 by anti-CCL2 antibody could reversed the increased M2 markers in MADCAM1^{D242N} reprogrammed-TAM.

(E) The GC cells co-cultured with MADCAM1^{P270Q}-reprogrammed or MADCAM1^{D242N}-reprogrammed-TAMs showed increased migration ability compared with control, which could be reversed by AKT inhibitor.

(F) The expression of CD274 (PD-L1), as well as some EMT markers (SLUG, MMP9), OCT4 and chemokines (IL4, IL13), were up-regulate in GC cell co-cultured with MADCAM1^{P270Q}-reprogrammed or MADCAM1^{D242N}-reprogrammed-TAMs, compared with GC cell co-cultured with MADCAM1^{WT}-reprogrammed-TAMs

Supplementary Figure 1

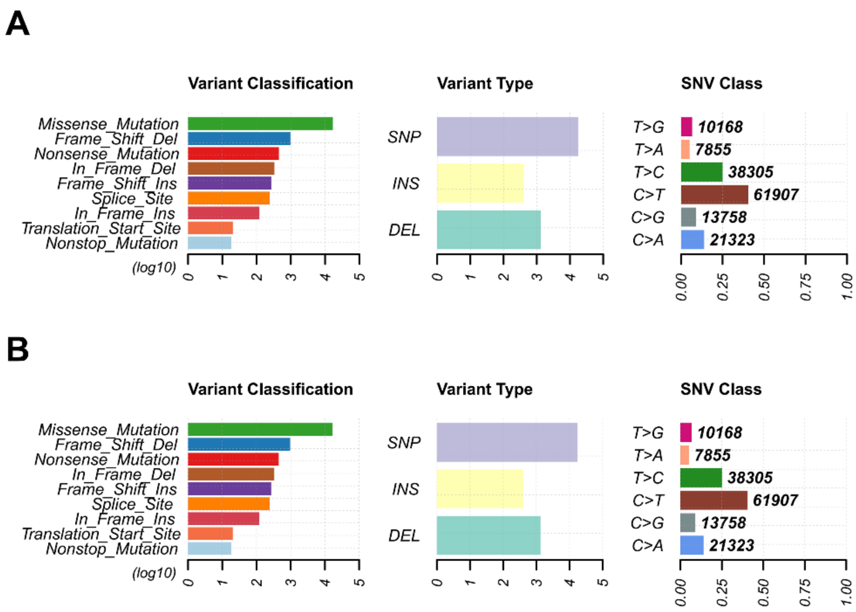
A
Variant Classification
Missense_Mutation
Frame_Shift_Del
Nonsense_Mutation
In_Frame_Del
Frame_Shift_Ins
Splice_Site
In_Frame_Ins
Translation_Start_Site
Nonstop_Mutation
(log10)
0 1 2 3 4 5
Variant Type
SNP
INS
DEL
0 1 2 3 4 5
SNV Class
T>G 10168
T>A 7855
T>C 38305
C>T 61907
C>G 13758
C>A 21323
0.00 0.25 0.50 0.75 1.00
B
Variant Classification
Missense_Mutation
Frame_Shift_Del
Nonsense_Mutation
In_Frame_Del
Frame_Shift_Ins
Splice_Site
In_Frame_Ins
Translation_Start_Site
Nonstop_Mutation
(log10)
0 1 2 3 4 5
Variant Type
SNP
INS
DEL
0 1 2 3 4 5
SNV Class
T>G 10168
T>A 7855
T>C 38305
C>T 61907
C>G 13758
C>A 21323
0.00 0.25 0.50 0.75 1.00

Supplementary Figure 2

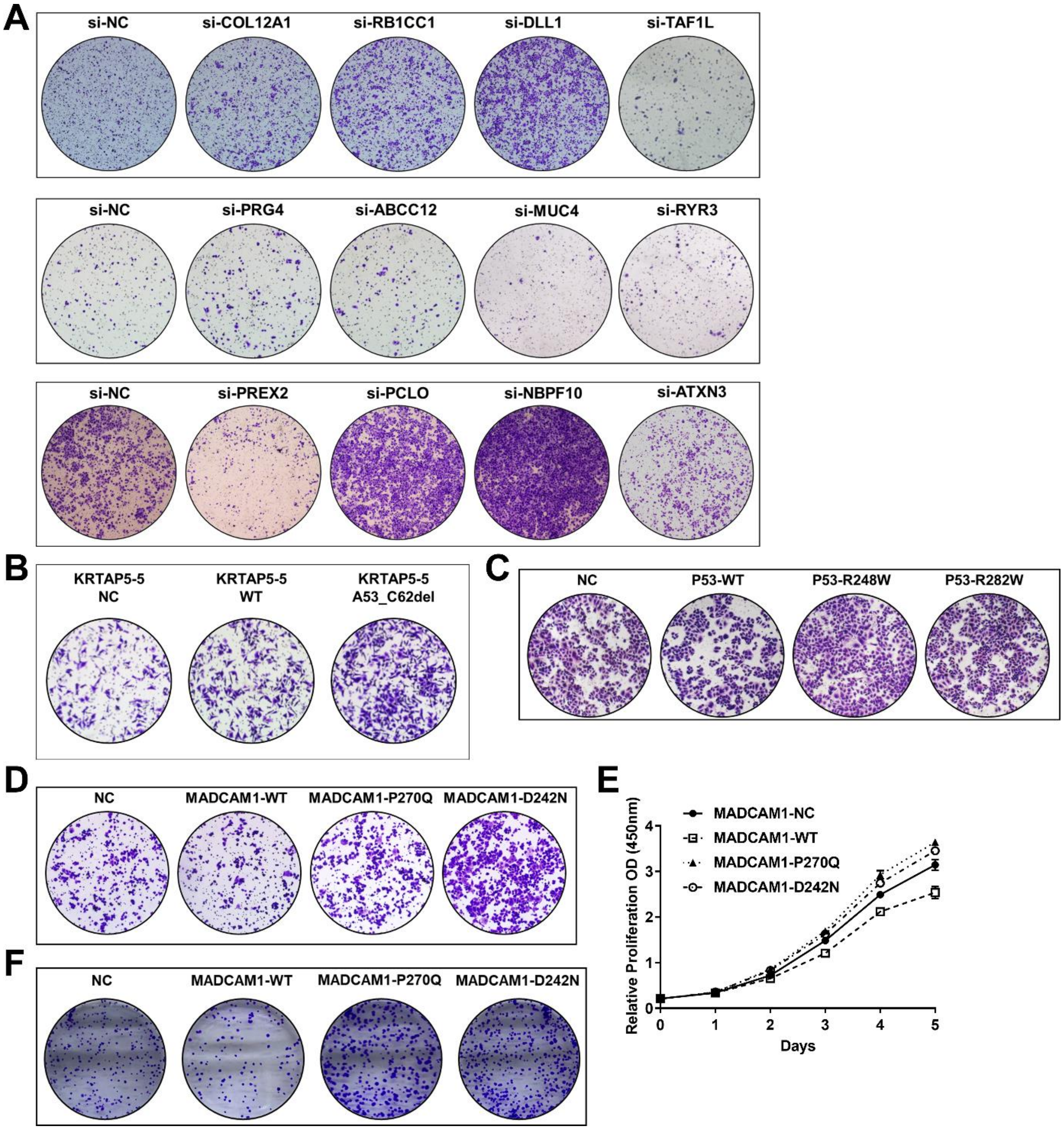

Supplementary Figure 3

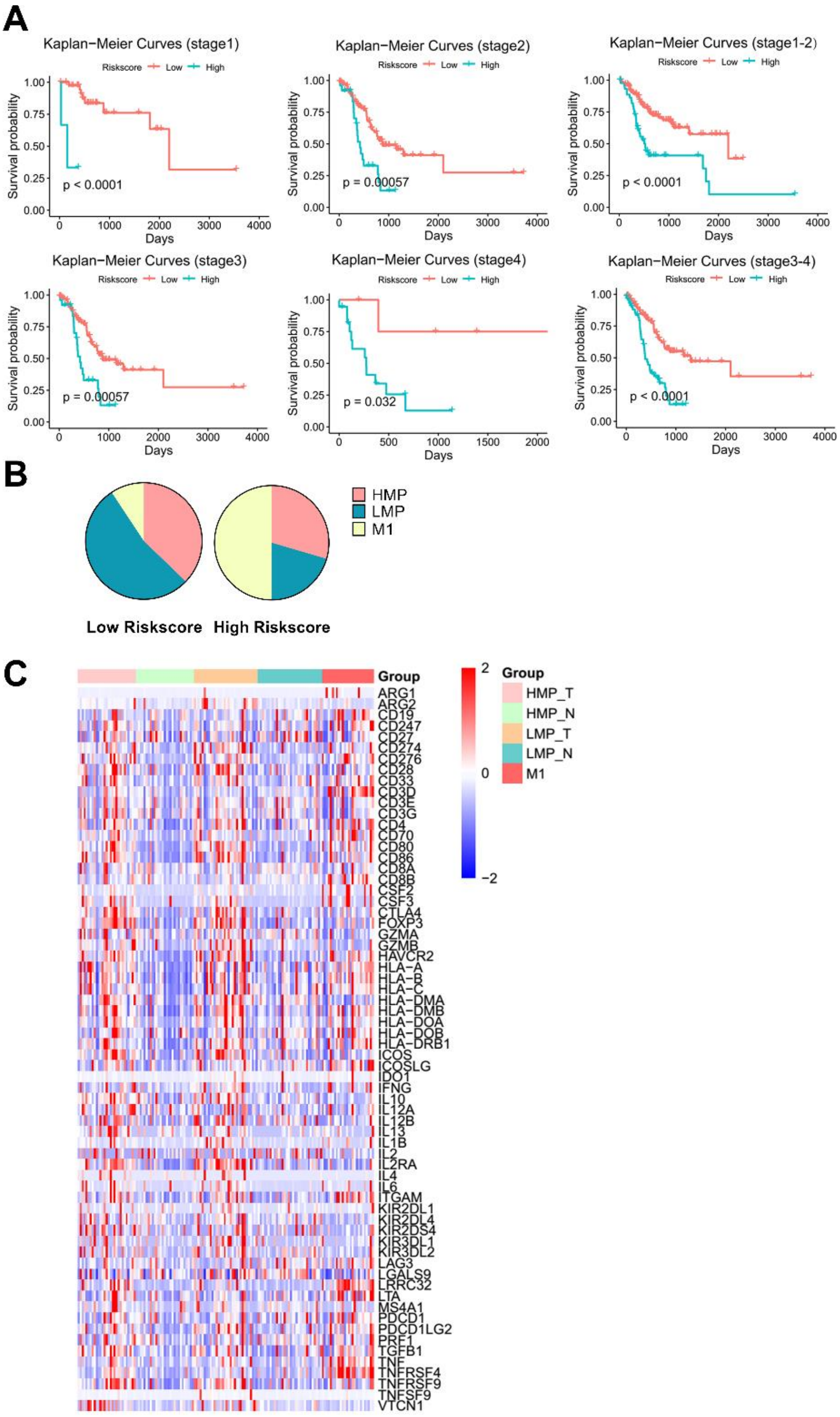

Supplementary Figure 4

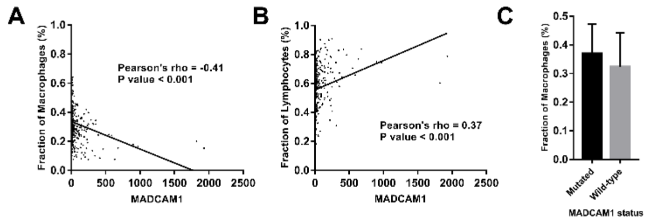

Supplementary Figure 5

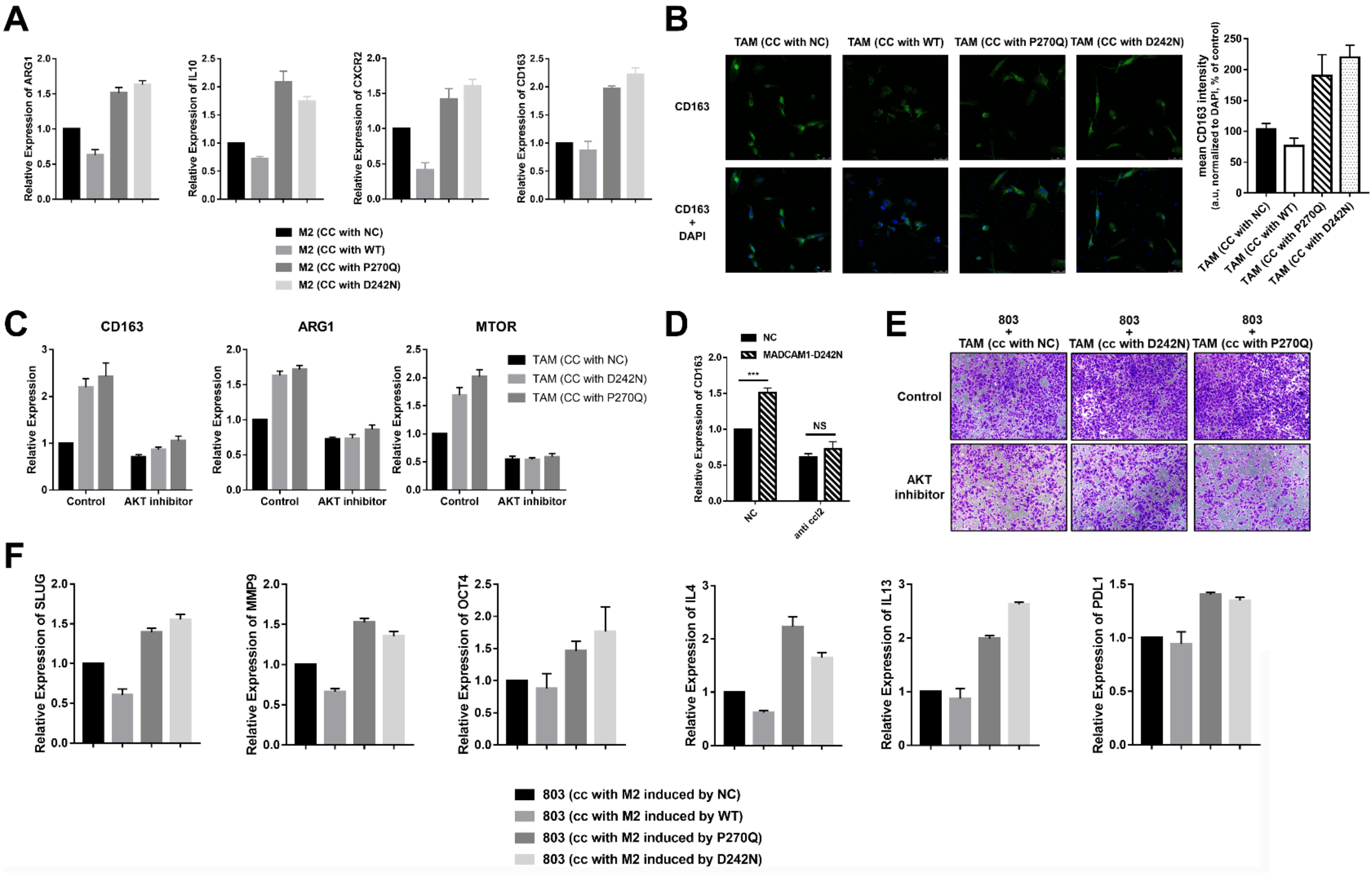

Supplement: Supplementary file 3 — Supplementary Figures 1-5 [file 41419_2022_4838_MOESM3_ESM.pdf]
